# Case of Eczema

**Published:** 1855-11

**Authors:** T. Bevan

**Affiliations:** Chicago


THE NORTH-WESTERN

MEDICAL AND SURGICAL JOURNAL.

NEW SERIES.

VOL. IV. NOVEMBER, 1855. NO. 11.

### ORIGINAL COMMUNICATIONS.

ART. I.—*Cases of Eczema, Reported to the Regular Meeting of the Cook County Medical Society, for October*, 1855. By T. BEVAN, M. D., of Chicago.

JUNE 19th, 1855.—Mr. Wm. Felley brought to my office his boy of 3 years, who had been affected with an eruption about the anus since the second week of his life. He had been treated in New York and this city, by various medical gentlemen, and with little or no amelioration of his symptoms—he presents the appearance of rather a debilitated condition of the general health, is somewhat emaciated though not very much so,—his bowels are alternately loose and constipated, appetite pretty good, very much annoyed by the eruption about the anus, which itches and pains him constantly. When he walks he straddles his legs wide apart so as to prevent the friction and consequent pain of the nates against each other. The eruption presents a continuous redish purple surface, presenting some points of excoriated surface, some veritable scabs and pustules of the kind appertaining to Eczema impetiginodes. Radiating from the circumference of the anus are numerous fissures varying in depth, from the 32d to the 1-16 of an inch, and length from ⅛ to ⅞ of an inch.

The history of the case as well as I could learn was, that the fissures had always existed; the redness and eruption was sometimes much better than at others, corresponding to the subsidence and desquamation of the Eczema. He has great pain at stool, especially if the fæces be hard or even of normal consistence, and his countenance presents the peculiar appearance of a person suffering from a constantly recurring pain.

The husband admitted to having had on two occasions the venereal disease; but from examination I was satisfied it had never made any constitutional appearance.—(He has a stricture also, which presupposed an antecedent gonorrhea. The parents are of the lower class of English—not particularly filthy, but of none the most careful habits.) The discharges from the bowels and bladder do not irritate by remaining in contact with the diseased surface. The whole surface diseased would amount to about ten or eleven superficial square inches.

My diagnosis was a case of chronic Eczema impetiginodes complicated with fissures of the anus. These fissures of the anus sometimes occasion the most tormenting pains, causing a sort of spasmodic action of the sphincter: judging, first, it was judicious to combat the eczema, I put the patient on the solution of arseniate of potassa. Fowler's solution, in doses of three drops, three times a day. To the surface of eruption, I directed to be applied after each discharge from the bowels a solution of sal soda, and regularly three times a day, cleansed with this solution, and an application of the solution of corrosive sublimate in the emulsion of bitter almonds. Applied at night after being well cleansed, I directed an ointment of

℞ Axunge, ℥j.

Ung. Hydrarg. ʒj.

Pulv. Opii. ℈j.

Tannin ʒj.

M. Ung.

To be freely applied to the surface. I should have stated that this, from the scales not being well removed at first, did not come in contact with the surface as I desired, and I directed a poultice of flax-seed meal, during two or three nights after which the above treatment was continued.

On the 25th, there was a very decided amelioration of the eczema; the surface was clean, and less pain with stools, the fissures were contracted,—some of them extending within the anus. Persisting, I directed the ointment to be introduced on the finger within the anus. The arsenic being borne well by the stomach, I directed five drops instead of three, at a dose, and the other remedies were continued, and when the bowels were constipated to be opened with flor. sulph. and magnes. sulph.

July 1st.—There has been rapid disappearance of the fissures; there is no pain at stool; appetite has been rather poor on account of arsenic causing some irritation of the stomach, diminished the dose to three drops, and continued medicine as before.

July 20th.—Almost well.

Sept. 1st.—The fissures have certainly disappeared, the skin has assumed the natural appearance, and the parts are perfectly sound and healthy. Child is rapidly increasing in flesh and vigor, and the cure is perfect.

---

CASE NO. II—Occurred in a girl of 11 years, the daughter of Mrs. W———l.

For ten months the scalp has presented points of inflammation characterized by small vesicles accuminated, confluent, agglomerated, and diffused over the surface of the scalp; containing a transparent serous fluid. These vesicles developed on a surface sometimes red and inflamed, and sometimes almost without redness or apparent inflammation of the skin, ruptured and exhale the liquid which is soon converted into white dry scales, adherent to the hairs and surface, amid which they are formed, and in this as in most cases of eczema of the scalp, swarming with small *deer* and their deposits.

The younger sister of the patient is affected with the disease, though not to the same extent as in the first case. The whole scalp, as far as there is hair, is attacked some with the coating of scales and vermin. I directed the hair to be clipped off as close as possible with scissors; a warm poultice of the linseed meal to be applied to the whole surface of the scalp, and following this the head to be thoroughly cleansed with the alkali. Against the vermin the unguentum hydrarg, and frequent cleansings of the scalp were directed ; following this the solution of

Corrosive Sublimate, grs. v.
Emuls. Bitter Almonds ℥j.
M.

Freely applied to the eruptive surface in conjunction with the arsenical solution, three drops at a dose, three times a day, and keeping the bowels free with the sulph. and magnes. Under this treatment the eczema rapidly disappeard, and six weeks from the commencement of the medication, the scalp was perfectly free from disease.

The other case of her sister was treated in the same way with the same results.

The results of these cases are very favorable to the effectiveness of the preparation of arsenic in this variety of chronic exanthemata, as a constitutional remedy ; and notwithstanding the fears that some entertain of its injurious effects on the system in the production of ascites, anasarca, etc., is one of our best remedies in combating those diseases. Cazenave, who has for years used the remedy in hundreds of cases, attests that when prudently administered it is not more dangerous than Quinine or a host of other remedies used every day in our ordinary practice.

Another remedy which was used in these cases, I would commend to the notice of the gentlemen of the society, which is the Emulsion of Bitter Almonds, either alone or holding in solution the Sublimate of Mercury. It is applicable to all cases of exanthematous diseases having a persistent tendency. There seems to be, especially with exanthemata, a local predisposition, as well as general, and in combating this I have found it especially useful, continued for months if necessary. I have seen it in several cases eradicate erysipelas of the face, and it is the only remedy known to me that will have this effect.

